# The Health Food Company

**Published:** 1876-12

**Authors:** 


Concerning the great work of this organization, we have a few diditional notes. They now exhibit :

GRANUATED BARLEY.

This is a very delicate and easily digested food, and is especially useful to persons of feeble digestive powers. The grain is the choicest white Barley, relieved of all hully trash by the careful processes made use of by this Company. As to nourishing properties, it stands next to Wheat, and above all the other cereals. Enriched with the Gluten, half and half, or alternated with it, it no donbt fulfills more perfectly the demand for a universal food than any other substance or combination.

This union of the best cereals we deem superior, as a food for infants, to any of the numerous advertised baby's foods and mixtures which are put up in packages, widely advertised, and sold at six times as much as they are worth. Infants who are being raised by hand, or who are about to be weaned, as well as all young children for whom good brains, good bodies, good teeth, good hair, and good physical and mental constitutions are desired, should be provided with such foods as the Health Food Co. are intelligently preparing. Mothers will take our advice and consult this scientific source rather than buy the mixtures of cracker-dust and bean-flour which are sold under various high-sounding names, and by the use of which permanent indigestion is often established, and the seeds of early death sown. We direct this Granulated Barley, or the mixture of Barley and Gluten, to be cooked like Rice. For very young childen, a gruel from these valuable substances should be made, to be strained and mixed with the best milk—half-and-half. The child who would not thrive on this food will not be saved by any other.

GRANULATED RYE.

This is a granular meal, from choice Rye, free from all insoluble hulls. All the Rye preparations we have ever seen till now have been pungent and bitter in taste. This is because Rye is a very dirty grain, accumulates much dust and acrid pollen, and is always more or less mixed with poisonous fungus known as ergot. All this dangerous stuff is removed by the processes of the Health Food Co., leaving a very delicate and nutty-flavored food. We advise our readers to try it as a bread and mush-making flour, as we are confident it will be employed by families to great advantage.

GLUTEN WATER.

These are the most nourishing cooked-food which can be made, because they are produced from the most nutritive substance known to chemistry —the pure gluten of White Wheat. The wafers are reasonably tender and crisp, and can be eaten by persons with imperfect teeth. They are certainly the most valuable cracker-food to be made, if not the best of any kind. As now made, they are quite palatable, and are certainly very easily digested.

CEREAL COFFEE.

The Health Food Co. have introduced a Cereal Coffee of a very nourishing kind, and just the thing for such persons as can not use tea or coffee without suffering from indigestion or nervous derangement. We use it at night in preference to other drink, because it seems to agree perfectly with the stomach, and to act as food as well as a pleasant and warming beverage.

